# Tailoring the Electron Trapping Effect of a Biocompatible Triboelectric Hydrogel by Graphene Oxide Incorporation towards Self-Powered Medical Electronics

**DOI:** 10.1021/acsbiomaterials.2c01513

**Published:** 2023-05-31

**Authors:** Andreia T. Pereira, Cátia R. S. Rodrigues, Ana C. Silva, Ricardo Vidal, João O. Ventura, Inês C. Gonçalves, André M. Pereira

**Affiliations:** † i3S − Instituto de Investigação e Inovação em Saúde, Universidade do Porto, 4200-135 Porto, Portugal; ‡ INEB − Instituto de Engenharia Biomédica, Universidade do Porto, 4200-135 Porto, Portugal; § IFIMUP − Instituto de Fisica de Materiais Avançados, Nanotecnologias e Fotónica, Departamento de Física e Astronomia, Faculdade de Ciências, Universidade do Porto, 4169-007 Porto, Portugal

**Keywords:** cytocompatible, energy harvesting, graphene oxide, hemocompatible, internet of medical things, poly(2-hydroxyethyl methacrylate), triboelectric nanogenerators

## Abstract

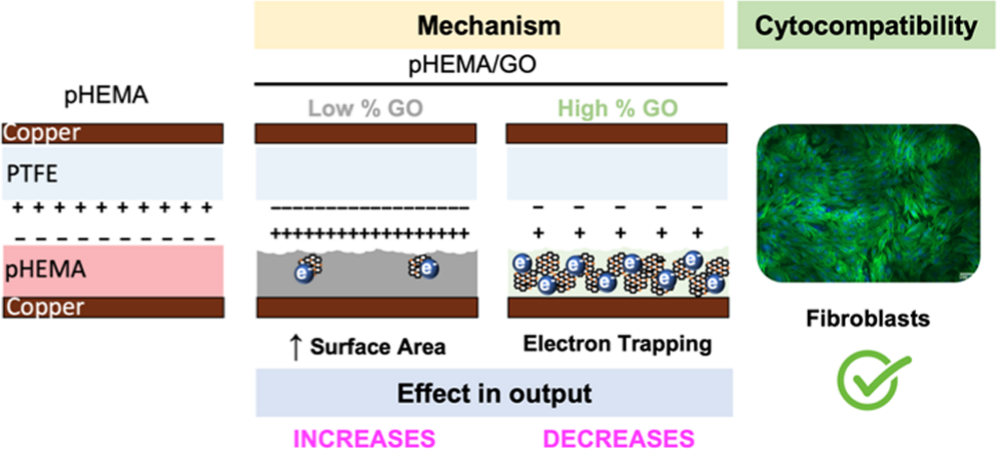

Triboelectric nanogenerators (TENGs) are associated with several drawbacks that limit their application in the biomedical field, including toxicity, thrombogenicity, and poor performance in the presence of fluids. By proposing the use of a hemo/biocompatible hydrogel, poly(2-hydroxyethyl methacrylate) (pHEMA), this study bypasses these barriers. In contact–separation mode, using polytetrafluoroethylene (PTFE) as a reference, pHEMA generates an output of 100.0 V, under an open circuit, 4.7 μA, and 0.68 W/m^2^ for an internal resistance of 10 MΩ. Our findings unveil that graphene oxide (GO) can be used to tune pHEMA’s triboelectric properties in a concentration-dependent manner. At the lowest measured concentration (0.2% GO), the generated outputs increase to 194.5 V, 5.3 μA, and 1.28 W/m^2^ due to the observed increase in pHEMA’s surface roughness, which expands the contact area. Triboelectric performance starts to decrease as GO concentration increases, plateauing at 11% volumetric, where the output is 51 V, 1.76 μA, and 0.17 W/m^2^ less than pHEMA’s. Increases in internal resistance, from 14 ΩM to greater than 470 ΩM, ζ-potential, from −7.3 to −0.4 mV, and open-circuit characteristic charge decay periods, from 90 to 120 ms, are all observed in conjunction with this phenomenon, which points to GO function as an electron trapping site in pHEMA’s matrix. All of the composites can charge a 10 μF capacitor in 200 s, producing a voltage between 0.25 and 3.5 V and allowing the operation of at least 20 LEDs. The triboelectric output was largely steady throughout the 3.33 h durability test. Voltage decreases by 38% due to contact–separation frequency, whereas current increases by 77%. In terms of pressure, it appears to have little effect on voltage but boosts current output by 42%. Finally, pHEMA and pHEMA/GO extracts were cytocompatible toward fibroblasts. According to these results, pHEMA has a significant potential to function as a biomaterial to create bio/hemocompatible TENGs and GO to precisely control its triboelectric outputs.

## 1 Introduction

With the rise of the digital era, the Internet of Medical Things (IoMT) appears as a breakthrough in the biomedical field. The IoMT consists of continuous monitoring of patients using sensors that promote data exchange between medical devices and health systems/services.^1−5^ The aim is to foster an accurate assessment and ultimately reduce the risk of medical errors and the associated healthcare costs.^1,2,5^ In addition, electronic medical devices (EMD) like pacemakers, left ventricular assist devices (LVAD), and brain stimulators have been extensively used to address various diseases.^6,7^ To power the sensors from IoMT and EMD and due to the mobility of patients, batteries are required.

Nonetheless, batteries still have a variety of constraints. Their need for replacement frequently entails surgical procedures, which lead to physical suffering for patient and financial costs for healthcare system. Furthermore, there is a danger of the batteries leaking their content into the surrounding tissue, posing a threat to patients.^8^ The large size and high weight of batteries restrict the miniaturization of EMD, which is one of the restraints for a broader implementation of IoMT. Accordingly, to enable the burst of IoMT and improve the performance of EMD, there is an intensive search for a clean, eco-friendly, endless, and safe electrical power source.^8−10^

Triboelectric nanogenerators (TENGs) convert random external mechanical energy into electrical power by contact–separation or relative sliding between two materials. The TENG principle is grounded in the conjunction of the triboelectric effect with an electrostatic induction. An electrostatic surface charge is created through contact between two triboelectric layers, which produces an electrical field to drive the electrons through an outer circuit. Differences in materials’ triboelectric polarities are essentially the drivers for the scavenging of mechanical energy from body movement, muscle contraction/relaxation, and cardiac/lung motions.^11^ Moreover, material features, such as porosity, have a high influence on the triboelectric outputs achieved. The first TENG device was developed in 2012 in Prof. Wang’s lab,^12^ and since then, several materials and multiple designs have been explored for a broad range of applications.^13−20^ Most materials used in TENGs cannot be applied in the biomedical field due to their cytotoxicity, immunogenicity, carcinogenicity, and/or thrombogenicity.^9,21,22^ Bearing these considerations foremost in mind, studies have recently emerged with the design of bio-TENGs that use natural, eco-friendly, degradable biomaterials,^23^ such as chitosan,^24^ starch,^25^ polylactic acid,^26^ cellulose,^27,28^ and gelatin,^29^ as reviewed in the literature.^9,30^

Herein, we explored an FDA-approved hemo/biocompatible hydrogel,^31^ poly(2-hydroxyethyl methacrylate) (pHEMA), as a nondegradable biomaterial for the development of a biocompatible and biostable TENG. As a hydrogel, pHEMA is made up of 60% water, resembling the composition of human tissues, which can prevent variation of electrical outputs in the presence of body fluids. The application of this hydrogel for energy harvesting had already been envisioned using the piezoelectric effect, reaching an output voltage of 15 mV under a compressive strain of 20%.^32^ In our previous studies, we showed that graphene oxide (GO), one of the strongest materials in the world, with a tensile strength of 120 MPa,^33^ can tune the mechanical properties of pHEMA, achieving hydrogels with 7.4- and 8.3-fold increased tensile resistance and stiffness, respectively,^34^ without compromising its hemocompatibility.^35^ The use of graphene-based materials has been explored to improve the performance of different nanogenerators.^36−38^ Xiu *et al.* showed that the incorporation of aligned graphene sheets in poly(dimethylsiloxane) (PDMS) increased its triboelectric outputs.^39^ From the literature, GO is negatively charged due to the presence of oxygen functional groups and exhibits negative tribopolarity.^40^ However, when incorporated in polymers, different outcomes are observed regarding the triboelectric properties. Huang *et al.* showed that in polyvinylidene fluoride (PVDF) nanofibers, GO acted as a charge trapping site, increasing the interface for charge storage and the output performance of TENGs.^41^ The incorporation of GO and the sodium dodecyl sulfate (SDS) surfactant in PDMS increases PDMS tribonegativity, generating a TENG with electric outputs 3-fold higher than those of the flat PDMS and delivering output voltage and current of up to 438 V and 11 μA/cm^2^, respectively.^42^ Parandeh et al. optimized a book-shaped TENG consisting of a layer of PCL/GO 4% fibers and a paper layer with the capacity to produce the maximum open-circuit voltage, short-circuit current, and load power of 120 V, 4 μA, and 116 μW, respectively.^43^ More recently, Ahmad et al. showed that tribonegative GO could enhance the tribopositivity of polyaniline through a new mechanism of disturbing the equilibrium state inside the tribopositive material under an impact force.^40^

Our study systematically evaluated the tribopolarity of pHEMA and pHEMA/GO composites containing different amounts of GO in an open circuit under contact–separation mode using PTFE as the reference triboelectric material. The achieved findings were correlated with the surface charge and topography of pHEMA and pHEMA/GO composites, revealing that GO can tailor the triboelectric properties by increasing the contact area of the triboelectric layer and acting as an electron trapping site. Materials’ cytocompatibility was assessed to forecast their potential application in the biomedical field.

## 2 Experimental Section

### 2.1 Synthesis of Graphene Oxide (GO)

Graphene oxide was obtained by the oxidation of graphite (purity: ≥99% and diameter: 7–11 μm, American Elements) through the modified Hummers’ method followed by its exfoliation, as described by us.^34,44^ Briefly, 3 g of graphite was added to 150 mL of a mixture containing H_2_SO_4_/H_3_PO_4_ (4:1). Then, the mixture was cooled to 0 °C and KMnO_4_ (18 g) was added to it. The reaction was stirred for 2 h and kept at 35 °C, followed by its cooling to 0 °C. 450 mL of distilled water was added slowly, and the excess of KMnO_4_ was eliminated by adding H_2_O_2_ until oxygen release stopped. After overnight resting, the material was washed by centrifugation at 4000 rpm for 20 min until the pH of the supernatant reached the pH of water (∼7). The suspension was sonicated for 6 h in an ultrasonic water bath to obtain GO and freeze-dried at −80 °C and 0.008 mbar for 3 days to obtain GO powder.

### 2.2 Production pHEMA/GO Composites

Poly(2-hydroxyethyl methacrylate) (pHEMA)/GO composites were produced by *in situ* polymerization of 2-hydroxyethyl methacrylate (HEMA) monomers as previously described by us.^34^ 0, 0.2, 0.4, 0.6, 1, 1.4, 5.5, 11, and 27.5% (v/v) of GO were added to a mixture of water/ethylene glycol (Sigma-Aldrich, 1.5 mL/2.25 mL) followed by the addition of 7.5 mL of the 2-hydroxyethyl methacrylate monomer (HEMA; >99.5%, Polysciences). To disperse GO, the mixture was sonicated in an ultrasonic water bath for 15 min. 0.345 mL of the cross-linking agent, tetraethylene glycol dimethacrylate (TEGDMA; Polysciences), and 1.5 mL of the redox initiator solution, which contained 20% ammonium persulfate (APS; 98%, Aldrich) and 7.5% sodium metabisulfite (SMB; 97%), were added to the mixture. Two clean glass plates with a 0.54 mm thick Teflon gasket were used as a mold, with the mixture being poured between them. The polymerization occurred overnight, and after that, hydrogels were released from the mold and soaked in distilled water for 4 h (water renewed every hour).

### 2.3 Characterization of GO and pHEMA/GO Composites

X-ray photoelectron spectroscopy (XPS) was used to evaluate the oxidation degree of GO. Briefly, the GO pellet (prepared in a manual hydraulic press) was analyzed using a Kratos Axis Ultra HAS (Kratos Analytical, U.K.). An Al monochromator with 15 kW was used as an X-ray source. The survey spectrum of GO was obtained at 80 eV and the C 1s high-resolution spectra at 40 eV.

The spectrum was deconvoluted using CasaXPS version 2.3.16, using Shirley’s background type. The C 1s spectral component was set at a binding energy of 284.6 eV to correct the contribution of the charge effect. The high-resolution C 1s spectra were fitted into seven peaks for the following binding energies: sp2 C=C (284.2–284.5 eV), sp3 C–C (284.8–284.9 eV), C–OH (285.3–286.0 eV), C–O–C (286.1–286.6 eV), C=O (287.5–287.9), O–C=O (288.8–288.9 eV), and π–π (290–292 eV). Due to its asymmetric nature, the sp2 carbon peak was fitted using an asymmetric Lorentzian function (LF) with an asymmetry parameter of 0.14. All of the other peaks were fitted with the Gaussian–Lorentzian (70: 30) function.

Transmission electron microscopy (TEM) of GO powders redispersed in water was performed to evaluate GO sheets’ exfoliation, lateral size, and morphology. The images were acquired using a JEOL JEM 1400 TEM (Tokyo, Japan) coupled with a digital camera (CCD Orious 1100 W, Tokyo, Japan).

The ζ-potential of GO in water was assessed by Laser Doppler Electrophoresis using a Malvern Zetasizer Nano ZS instrument (Malvern Instruments Ltd, Worcestershire, U.K.). For these measurements, the samples were dispersed in water.

Scanning electron microscopy (SEM) was performed to visualize the surface morphology of pHEMA and pHEMA/GO composites. The materials were dried in a vacuum oven at 60 °C and coated with a thin layer of gold/palladium by sputtering to improve the samples’ conductivity. An FEI Quanta 400 FEG ESEM/EDAX Genesis X4M SEM with accelerating voltages of 15 kV (GO) and 10 kV (pHEMA/GO composites) was used to visualize the samples.

Captive-bubble contact angles of hydrated pHEMA and pHEMA/GO composites were evaluated using the inverted drop method in a DataPhysics goniometer, model OCA 15, equipped with a video CCD camera. For this, the samples were placed in a glass chamber with ultrapure water and individually attached to steel slides. A 10 μL air bubble was released from a J-shaped needle onto the surface of the sample, and the contact angle was then calculated using SCA software.^35^

The ζ-potential of pHEMA/GO films was determined from streaming potential measurements with a commercial electrokinetic analyzer (EKA, Anton Paar GmbH, Austria) using a rectangular cell for small flat samples with a variable channel height. One sample (2 × 1 cm^2^) was glued on each poly(methyl methacrylate) (PMMA) block and mounted in parallel on each side of the cell, creating a rectangular (2 × 1 cm^2^) slit channel between the sample surfaces. The height of the slit channel was maintained constant for all of the measurements using a micrometer screw, which was adjusted after checking the flow in each direction. The streaming potential was measured using Ag/AgCl electrodes installed at both ends of the streaming channel. The electrolyte used was 1 mM KCl with a pH of 5. The experiments were performed at room temperature. The conductivity of the electrolyte solution was measured during the assay. The streaming potential was measured while applying an electrolyte flow in alternating directions and ramping the pressure from 0 to 400 mbar.

### 2.4 Cytocompatibility of pHEMA/GO Composites

The cytocompatibility of pHEMA and pHEMA/GO composite extracts was evaluated toward human fibroblast HFF-1 cells according to ISO 10993-5:2009(E). Material extracts were prepared as described in ISO 10993-12:2012,^45^ namely, discs of pHEMA and pHEMA/GO composites with Ø = 13 mm sterilized with ethanol 70% (v/v) and rinsed with PBS. The extracts were obtained by incubation of DMEM culture medium (Dulbecco’s modified Eagle’s medium (DMEM, Gibco) supplemented with 10% (v/v) fetal bovine serum (FBS, Gibco) and 1% (v/v) penicillin/streptomycin (Biowest)) with the materials for 24 h at 37 °C in an orbital shaker at 100 rpm. The cells were seeded in 96-well plates at a density of 1 × 10^5^ cells/mL (100 μL) and maintained in culture for 24 h in DMEM+. After that, the culture medium was replaced by material extracts. After 24 h of incubation at 37 °C, the mitochondrial metabolic activity of the cells was quantified by the resazurin assay. Extracts from TCPET discs were used as a positive control of cell growth, while a solution of DMEM with 1 mM H_2_O_2_ was used as a negative control. Assays were performed with *n* = 5 and repeated twice.

### 2.5 Triboelectric Properties of pHEMA/GO Composites

To measure the generated electrical signals, copper tape (1.5 × 2 cm^2^) was attached to triboelectric materials (1.5 × 2.5 cm^2^) to serve as electrodes and fixed on glass plates working as substrates.^46^ On one side, PFTE was used as the reference material, and on the other side, pHEMA or pHEMA/GO (0–27.5% v/v) was attached. A homemade systematic testing system made the two triboelectric materials come into contact. The generated voltage, current, and power were measured as a function of the load resistance using a circuit board with resistors from 100 to 1 GΩ. Furthermore, a diode bridge was used to convert alternating current (AC) to direct current (DC) to charge a 10 μF capacitor^46,47^ and turn on light-emitting diodes (LEDs) to monitor the operation of the TENG.

Under room temperature, the impact of contact–separation factors was tested by modulating the pressure (20–60 psi), frequency (0.3–2 Hz), and lifespan up to 12 000 cycles (room temperature and humidity).

## 3 Results and Discussion

### 3.1 Materials Characterization

GO was obtained by the chemical oxidation of graphite by the modified Hummers’ method, followed by mechanical exfoliation in an ultrasonic bath.^34^

According to XPS analysis, the synthesized GO is composed of 66.8% carbon and 33.2% oxygen (Figure S1A). Epoxides (C–O–C) represent 42.2% of all functional groups present in GO, making them the most prevalent oxygen-containing group. The remaining two oxygen-containing groups are carbonyl (C=O), with 11.0%, and carboxyl (COOH), with 3.7%. Regarding morphology, GO platelets exhibit a wrinkled structure and appear mainly as a single layer or few layers when dispersed in water, as can be seen by TEM images (Figure S1B). These morphological features are specific to oxidized forms of graphene materials, since the presence of oxygen-containing groups in the platelets promotes the establishment of hydrogen bonds with each other and/or with water molecules, which leads to platelet folding and/or an improved dispersion, respectively. GO has a ζ-potential of −33.0 ± 1.3 mV, which is typical for the oxidized forms of graphene.^48^ This negative charge is related to the strong presence of oxygen-containing groups on the GO sheets, particularly carboxyl groups that tend to deprotonate in aqueous media.

**Figure 1:**
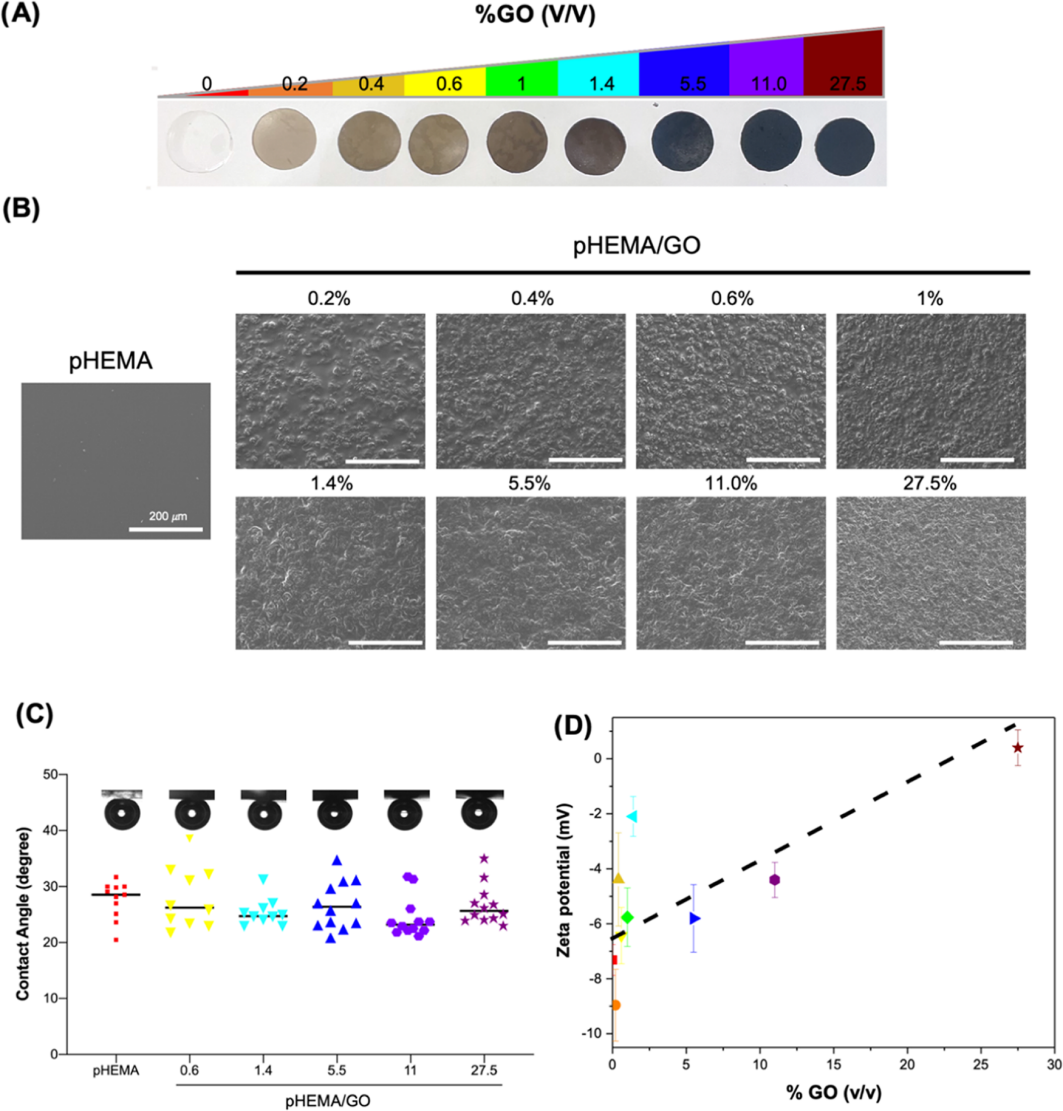
Surface features of pHEMA and pHEMA/GO composites. (A) pHEMA and pHEMA/GO hydrogels, (B) SEM images, (C) captive air bubble contact angles, and (D) ζ-potential as a function of GO amount incorporated in the formulation.

GO incorporation changes the color of pristine pHEMA, which is transparent, toward brown, where an increasing gradient is seen depending on the GO amount (Figure 1A). SEM images (Figure 1B) show that the surface tends to become rough with the level of GO.^34^ At even small concentrations (0.2% v/v), this is evident. Despite the observed increase in surface roughness, incorporation of GO in pHEMA does not change its captive air bubble contact angles, which remain ∼29°, demonstrating that surface hydrophilicity is unaffected (Figure 1C). This effect was previously observed for pHEMA^34^ and PEG^50^ hydrogels.

Figure 1D depicts the ζ-potential of pHEMA/GO composites as a function of the GO content in pHEMA. Neat pHEMA hydrogels exhibit a negative ζ-potential of −7.3 mV (pH ∼ 5). This can be attributed to polar moieties, namely, the hydroxyl and ester groups, in the pHEMA chemical structure. As the GO content in the formulations increases, the ζ-potential appears to increase linearly, reaching 0.4 mV for 27.5% (v/v). This effect was distinct from that reported in the literature, where the GO incorporation into polyvinylidene difluoride (PVDF),^48^ PVDF/poly(vinyl pyrrolidone),^48^ poly(vinyl alcohol),^51^ and chitosan^52^ leads to a decrease in the polymers’ ζ-potential due to the contribution of the negative charge of GO. In pHEMA, HEMA monomers can adsorb onto the GO surface,^35^ implying that HEMA covers GO before hydrogel polymerization. Such interaction is probably achieved by establishing hydrogen bonds between the polar moieties of HEMA and GO; hence, these groups are less exposed at the composite surface. This could explain the unexpected increase of the pHEMA ζ-potential upon GO incorporation. Despite this, we previously showed that pHEMA surface hydrophilicity (around 55°) does not change upon GO incorporation.^34,35^

### 3.2 Cytocompatibility

**Figure 2:**
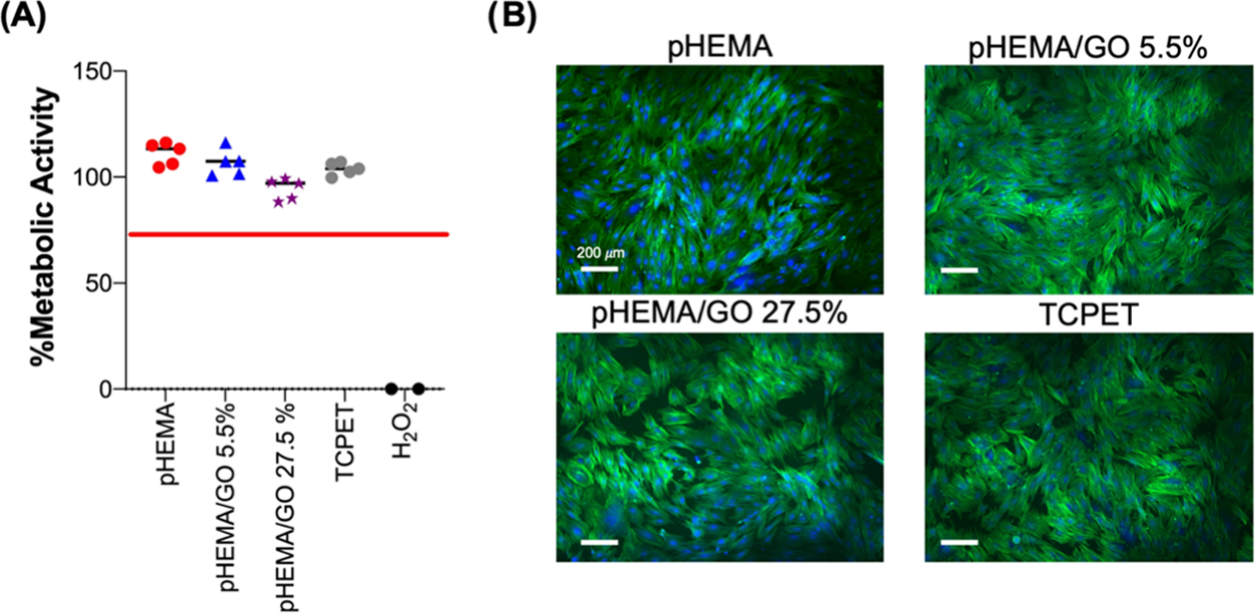
Cytotoxicity of the pHEMA and pHEMA/GO composite extracts. (A) The metabolic activity of the cells is represented in percentage compared to cells growing in DMEM+ (100%; 70% of the metabolic activity is the lower limit to consider the material extracts’ noncytotoxicity (red line)). (B) Cell morphology of HFF-1 after 24 h incubation with the material extracts (DMEM+ as the extraction vehicle). Extracts from TCPET discs were used as a positive control and 1 mM H_2_O_2_ (in DMEM+) as a negative control.

The cytocompatibility of pHEMA and pHEMA/GO (5.5% and 27.5%) hydrogels was evaluated according to ISO 10993-5:2009(E), using materials extracts tested toward human foreskin fibroblasts (HFF-1). The results showed that none of the obtained extracts affects the morphology and/or metabolic activity of fibroblasts (Figure 2), confirming the lack of cytotoxicity. These results corroborate the previously reported data that revealed that pHEMA and pHEMA/GO extracts were cytocompatible toward rat fibroblasts and human endothelial cells.^34^

### 3.3 Triboelectric Properties

**Figure 3:**
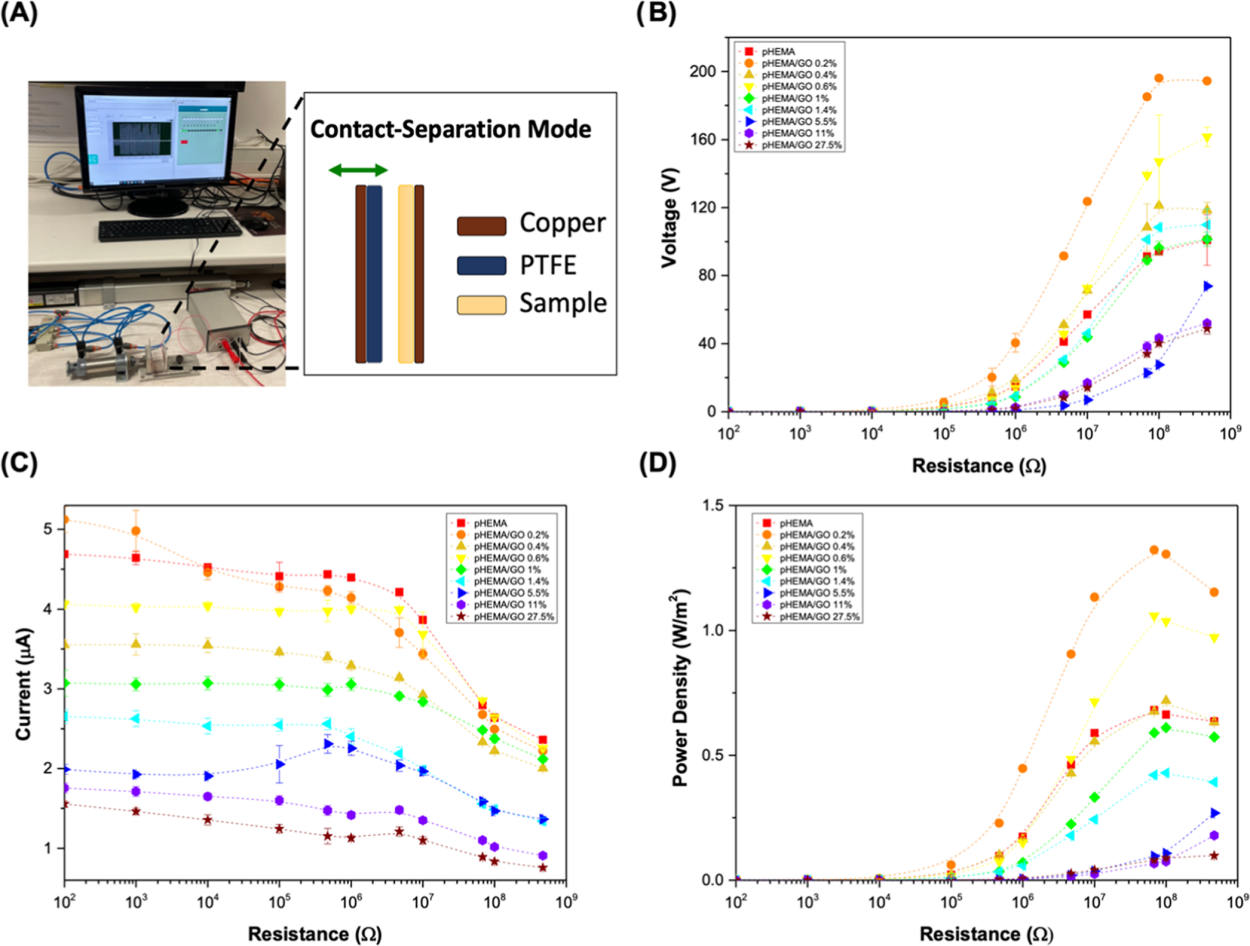
(A) Schematic illustration of the triboelectric contact–separation mode measuring setting; (B) open-circuit voltage; (C) short-circuit current; and (D) power density of neat pHEMA and pHEMA/GO composites.

The triboelectric properties of pHEMA and pHEMA/GO composites were evaluated in contact–separation mode, using PTFE, a tribonegative material, as the reference (Figure 3A). Figure 3B–D shows the generated short-circuit voltage, current, and power density for all tested materials.

For pHEMA and pHEMA/GO with GO concentrations up to 5.5%, the behavior of voltage curves as a function of resistance is similar, presenting a typical S-shape, with outputs starting to rise for resistances of 10^5^ Ω and reaching saturation at 100–470 MΩ. Signal saturation was not reached for the remaining concentrations within the resistance range applied. In terms of the current, it can be seen that all formulations exhibit the same curve profile (inverted S-shape like), where current outputs are constant until the resistance reaches 10^6^ Ω, at which point it starts to decrease (Figure 3C). Since the power density is derived from the outputs of voltage and current, both parameters have an impact on its curve profile. As a consequence, for 0.2–5.5% pHEMA and pHEMA/GO, the maximum power density was achieved for a resistance of 68 MΩ, while for higher GO concentrations, it was not possible to achieve (Figure 3D). Although it is conceivable to use higher resistances to reach missed saturation points, all measurements are always constrained by the electrometer’s internal resistance.

Regarding the triboelectric outputs, for the pHEMA counterpart, a voltage of 100.0 V under an open circuit, a current of 4.7 μA, and a power density of 0.68 W/m^2^ for an internal resistance of 10 MΩ are the highest output parameters.

**Figure 4:**
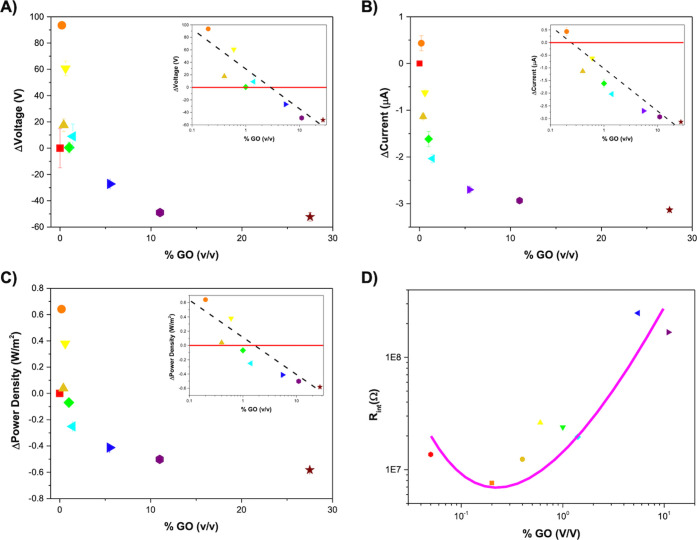
Output variation upon GO incorporation compared with neat pHEMA: (A) open-circuit voltage, (B) short-circuit current, and (C) power density. (D) Internal resistance as a function of GO concentration.

The variation of the voltage (ΔVoltage), current (ΔCurrent), and power density (ΔPower Density) relative to the pHEMA output counterpart will be determined in order to assess the overall output performance changes shown in Figure 3. These variation results are displayed in Figure 4A–C.

Findings on ΔVoltage demonstrate the presence of two regimes: one in which all of the variations are positive in the concentration range < 2.6% (low % GO). Likewise, all of the variations are negative in the high-concentration zone (>2.6%; high % GO). The smallest concentration, 0.2% (v/v), is the greatest value for ΔVoltage = 94.5 V. Above this concentration, the variations start to decrease as % GO increases logarithmically (see the insets of Figure 4A), inverting the signal for concentrations above 2.6%. This logarithmic variation leads to the establishment of a plateau for ∼ 10% v/v GO, leading to a ΔVoltage of −48.9 V.

Similar behavior to ΔVoltage is observed in the case of ΔCurrent, although the inversion occurs for lower concentrations > 0.2% (v/v), meaning that only the sample with 0.2% reaches a positive variation (ΔCurrent = 0.5 μA). Above this, the current also drops with a quasi-linear tendency like in the case of the voltage reaching the lowest value of ΔCurrent (−2.9 μA) for 27.5% (see the inset of Figure 4B).

Concerning the power density, since it depends on the voltage and current, a similar behavior is observed, reaching a maximum ΔPower Density of 0.68 W/m^2^ for 2.6% and then decreasing until reaching a plateau for 27.5% with a ΔPower Density of −0.6 W/m^2^ (see the inset Figure 4C).

It is noteworthy that a further increase in the GO amount does not affect the output performance possibly due to GO aggregation, as observed before when GO was incorporated in polyaniline (PANI)^40^ and PDMS.^42^

Figure 4D depicts the internal resistance of the pHEMA and pHEMA/GO composites, which was determined by the resistance where the current and voltage curves intersected. The incorporation of 0.2% GO into pHEMA results in a decrease in internal resistance from 14 to 7.6 MΩ, whereas the incorporation of 5.5% GO leads to a substantial increase of 25 MΩ. Nevertheless, the internal resistance for the highest tested formulation, 27.5%, is higher than the range of resistances available in our experimental setup.

**Figure 5:**
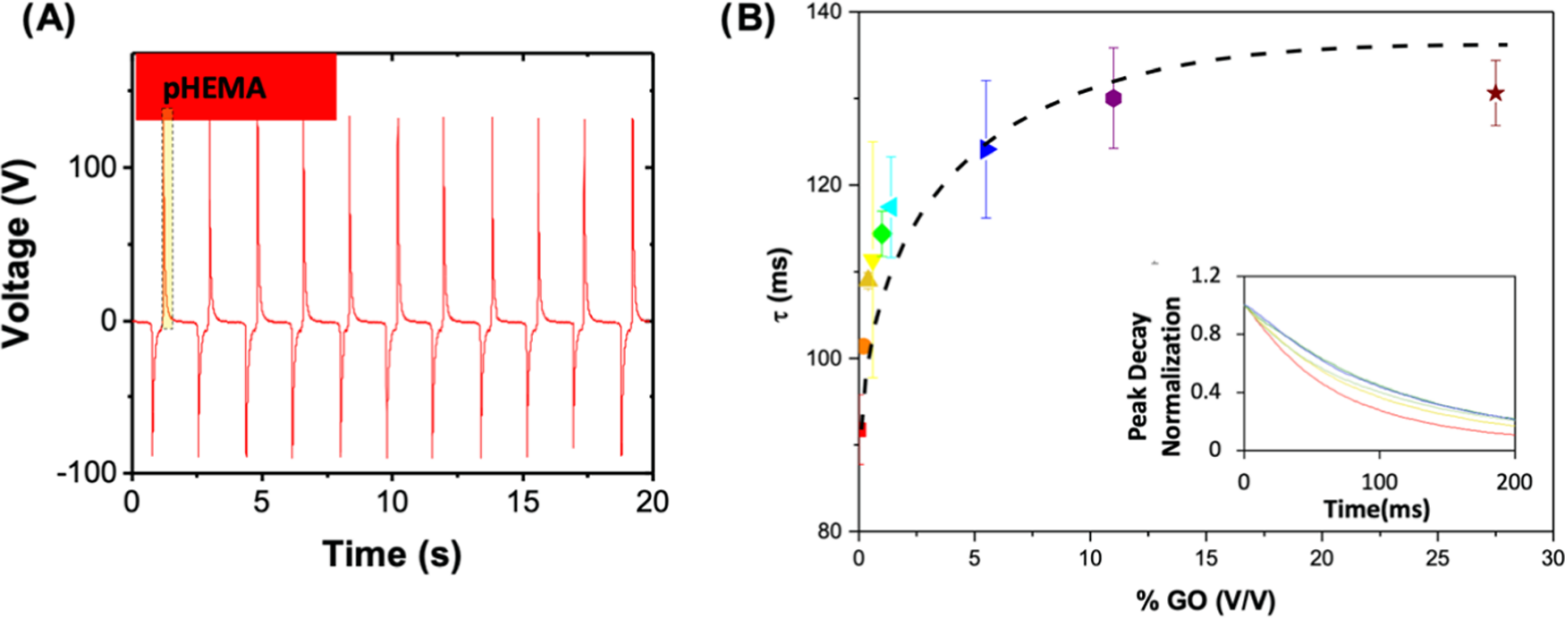
Analysis of pHEMA’s open-circuit tension. (A) Schematic representation of the analyzed part. (B) τ of peak dropping.

Considering that TENGs can inherently have capacitor behavior,^53^ a detailed analysis of the open-circuit tension was performed to study the charge decay characteristic time (τ). This analysis was made at the ascending peak, namely, between the positive peak current and maximum compression (Figure 5A). The normalized peak decay curve for the various GO concentrations in order to be independent of the voltage intensity is shown in the inset of Figure 5B. As the concentration of GO increases, there is a delay in peak decay. This is related to a change in the charge decay characteristic time. By fitting these curves, τ was determined and is depicted in Figure 5B. The decay time (τ) increases with the amount of GO, from 90 to 120 ms. Note that this metric increases uniformly as GO increases, in contrast to current and voltage where a maximum is observed for 0.2% and then a decrease in the outputs was observed.

These differences between τ and ΔVoltage and ΔCurrent suggest that two mechanisms control the output performance when changing the GO amount.

By embedding GO into the pHEMA matrix, the SEM indicates an upsurge in surface roughness, which increases the contact surface area and carrier concentration and is thus expected to increase the voltage output. This mechanism could explain the low GO % regime where a positive variance in voltage is obtained. Moreover, the same SEM pictures reveal that the roughness tends to stabilize around 2.6%, hence the contact surface area should stay the same for high percentages. However, the fact that the sign of ΔVoltage and ΔCurrent changes at different GO% values is a result of an additional mechanism occurring in the pHEMA matrix when GO% is added.

The shifting permittivity of the medium is one explanation that could be used to explain the behavior. pHEMA has a value of ∼28,^54^ but GO has a range of 30–50.^55^ Hence, the addition of GO would result in an increase in permittivity, which would then increase the surface charge, current, and voltage.

But nonetheless, the reverse of this impact is actually seen. As a result, another phenomenon must be taking place. Recently, it was shown by molecular simulations that GO has a high affinity to capture electrons on its structure, acting as charge trapping sites in composites.^56^ Their study showed that the oxygen-containing groups, C–O–C, COOH, and C–OH (when in the middle of the GO sheet), are the key players in capturing these electrons, since their electron capture energy is 0.16 eV higher than that of the intact graphene structure. As shown by XPS data, the GO synthesized in our study has high amounts of these oxygen-containing groups in their composition, 45.9% (C–O–C and COOH), which corroborates the observed electron trapping effect. As such, electrons attracted from the contact–separation of PTFE and pHEMA/GO were stored either in the discrete, quantized levels of these nanosized graphene particles or trapped in the amorphous GO dielectric. Moreover, the relaxation time variation indicates a deceleration of surface charge dynamics, which constitutes a fingerprint of the electron trapping effect triggered by the GO presence in the pHEMA matrix.

This electron trapping effect promoted by the presence of GO was previously reported when GO was inserted in the polyaniline polymer, resulting in an enhancement in tribopositivity,^40^ PVDF nanofibers,^57^ and epoxy resin.^56^

This hypothesis is supported by the observed increases in pHEMA’s surface ζ-potential and internal resistance within the incorporated amount of GO. Since GO is embedded in the pHEMA matrix, its ability to scavenge electrons turns the pHEMA surface less negative (Figure 1D), which results in an increase in the surface ζ-potential. Furthermore, the GO’s trapping effect reduces electron velocity within the pHEMA matrix, increasing its internal resistance (Figure 4D).

**Figure 6:**
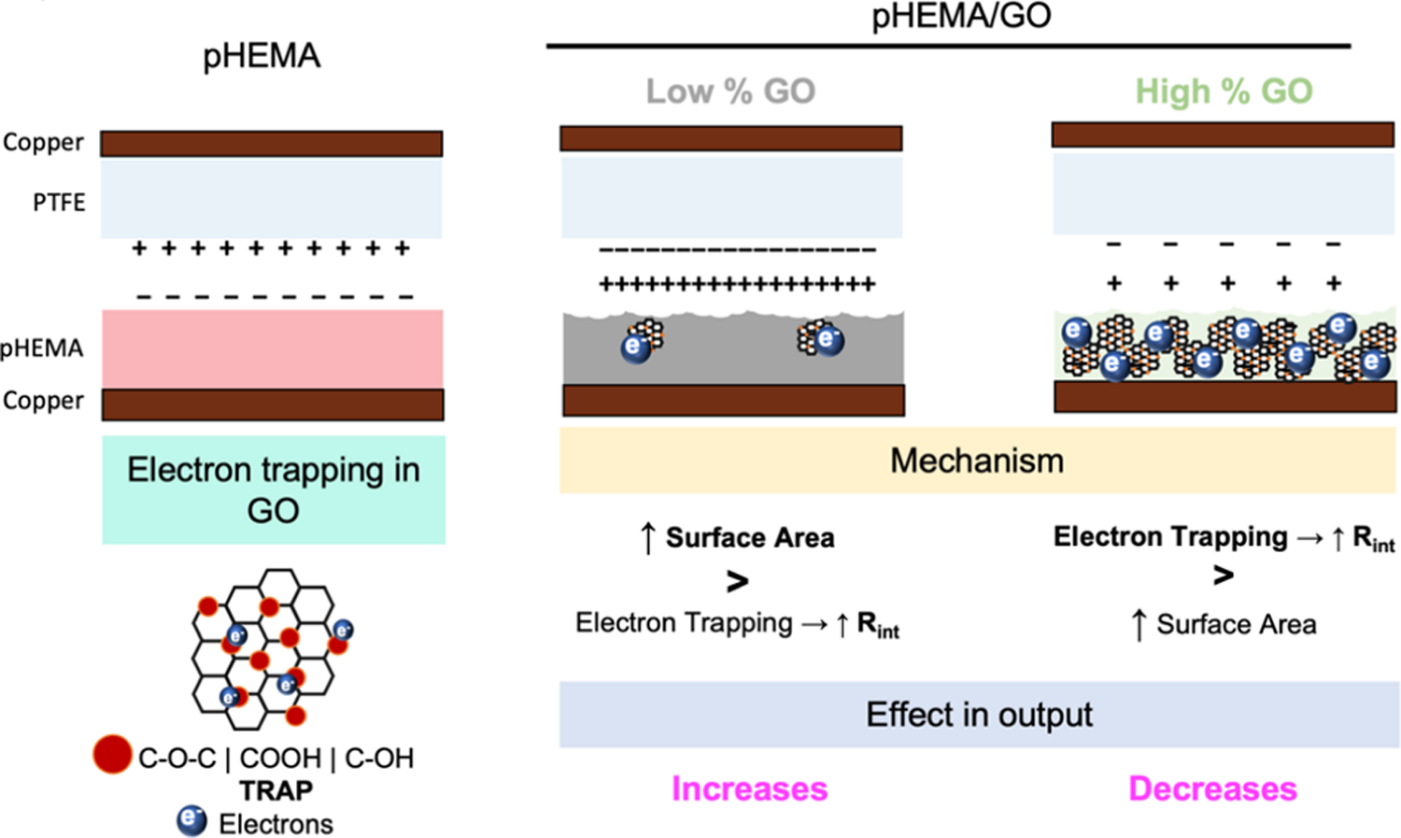
Schematic representation of the mechanism associated with the tunning of pHEMA’s triboelectrical properties upon GO incorporation.

Taking into account all of our findings, Figure 6 depicts our proposed mechanism for describing the observed effects on pHEMA triboelectric properties after the GO incorporation. When GO is incorporated into pHEMA, two major players contribute to the achieved triboelectric outputs: surface area increase and GO’s electron trapping effect. As a result, when GO is incorporated into low amounts, the increase in surface area outweighs the electron trapping effect in pHEMA, resulting in an increase in triboelectric output (see Figure 1B). The electron trapping effect is more pronounced at higher amounts of GO, where no additional changes on the surface morphology are observed, making it impossible to compensate with the observed increase in surface area, which leads to a decrease in triboelectric output.

Considering the lack of cytotoxicity and the non-fouling properties towards cells, platelets, and bacteria,^35^ both pHEMA and pHEMA/GO show high potential to develop TENG for the biomedical field, in particular for implantation. Depending on the application and desired outputs, different amounts of GO can be used to tune the triboelectric properties of pHEMA. The use of pHEMA and pHEMA/GO as triboelectric pairs can also be envisioned, since they could have different tribopolarities.

### 3.4 Energy Storage and Durability Tests

**Figure 7:**
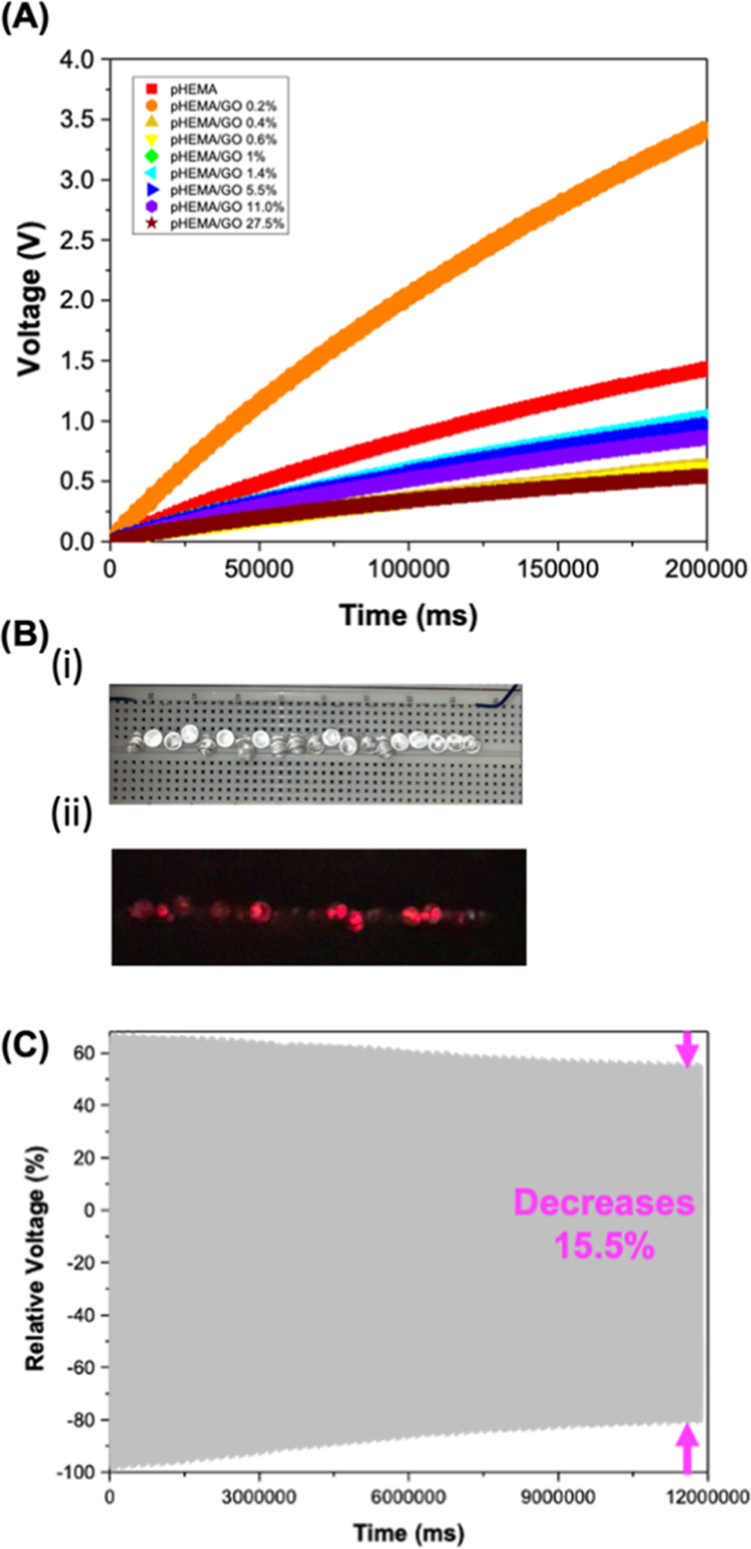
(A) Charging voltage over time of the 10 μF capacitor with triboelectric pairs of pHEMA or pHEMA/GO composites versus PTFE, (B) lighting up 20 red LEDs (i) off and (ii) on with the triboelectric pair pHEMA/GO 1% versus PTFE. (C) Relative voltage during 3.3 h of the durability test.

The capacity to store the energy generated by triboelectric pairs comprising neat pHEMA or pHEMA/GO composites with PTFE in a 10 μF capacitor was evaluated. For this, the terminals of the setting were connected to a full bridge diode rectifier that, in turn, was connected to a load capacitor.^47^Figure 7 shows that all triboelectric pairs can charge the 10 μF capacitor even though they are not able to saturate it during the testing time (200 s). For pHEMA, the capacitor was charged up to 1.5 V. After incorporating 0.2% GO in pHEMA, it was possible to increase the charging voltage of the 10 μF capacitor to 3.5 V. For the higher concentrations of GO, the charging voltage decreased to 0.25 V. These results corroborate the proposed mechanisms for the tailoring effect of GO on pHEMA’s triboelectric properties.

In addition to the ability to store energy in capacitors, the number of LEDs that can be lit by TENGs is a major concern in the development of nanogenerators, as it also illustrates the usefulness of the energy generated. Our results show that up to 20 red LEDs could be lit using the triboelectric pair pHEMA/GO 1% versus PTFE.

In the vertical contact–separation mode of TENGs, mechanical abrasion during the cycles can damage the triboelectric layer and reduce the generated outputs.^58^ Considering this, durability tests were performed for the triboelectric pair pHEMA/GO 1% versus PTFE for 3.33 h. Our findings show that even after 12 000 cycles, the output voltage remains almost similar, with only a slight decrease of 15.5%. Moreover, the highest decrease is observed in the first 6000 cycles, where the voltage outputs decrease by 11% and remain almost similar for the rest of the durability test.

### 3.5 Influence of Frequency and Pressure on Triboelectric Outputs

**Figure 8:**
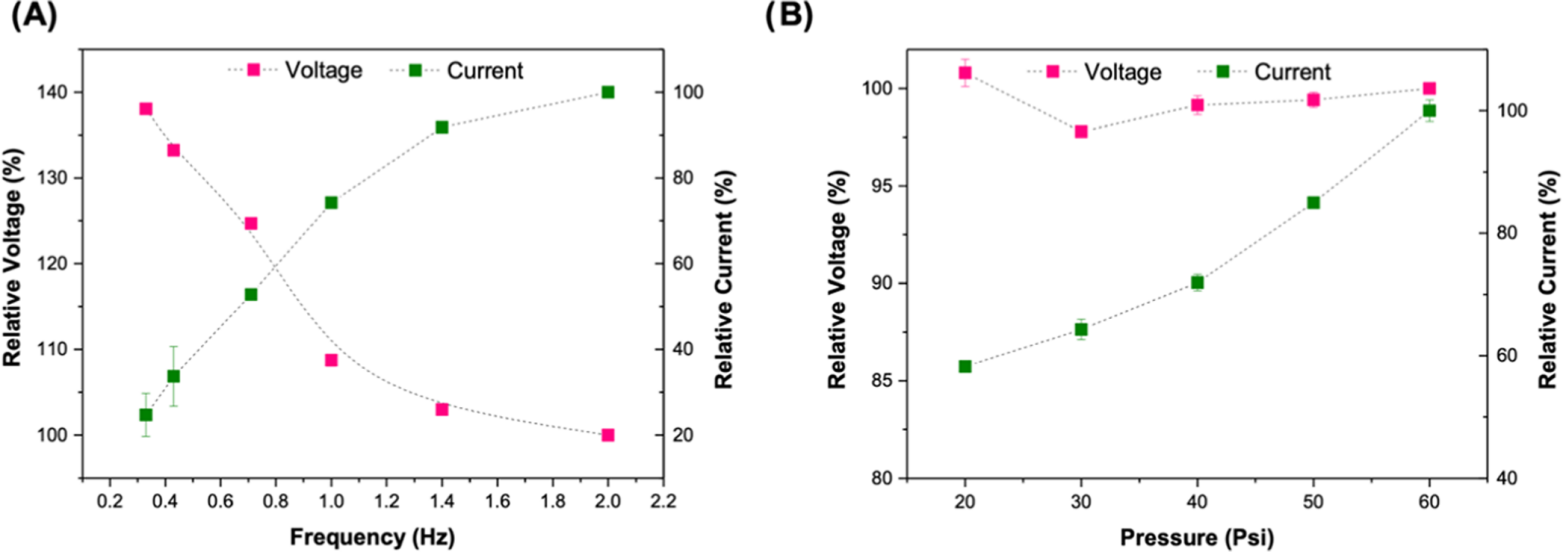
Influence of (A) frequency and (B) pressure in obtained voltage and pressure with triboelectric pair pHEMA/GO 1% vs ePTFE. Relative voltage and current were determined considering 2 Hz and 60 Psi the 100%.

To simulate the normal heartbeat of an adult at rest, contact–separation cycles were performed in all of the prior tests at a frequency of 1 Hz. Nonetheless, it is well known that frequency can have an impact on the generated triboelectric output. As a result, we investigated how frequency and pressure affected the triboelectric outputs. Figure 8A demonstrates how the output voltage can decrease up to 38% as the frequency increases, while the current can increase up to 77%. Ishara *et al.* previously showed that for TENGs, the generated power output increased as the frequency increased.^59^ A threefold increase in pressure causes a 42% increase in current output despite appearing to have no effect on voltage output (Figure 8B). This is a predictable result, since it is anticipated that increasing the pressure will boost the charge density at the surface, which increases the output. This effect was previously reported for other TENG works.^60^

## 4 Conclusions

A scalable and cheap method was proposed to achieve biocompatible materials with different tribopolarities to develop TENGs. The incorporation of GO in pHEMA can be used to tailor an increase in surface roughness, charge, and internal resistance. In terms of the triboelectric effect, GO increases the output by increasing the surface area at low concentrations, while at high concentrations, GO’s electron trapping capacity causes a decrease in triboelectric output. The energy produced by all materials enables the charging of a 10 μF capacitator, showing the possibility of storing this electrical energy and lighting up at least 20 LEDs. During 3.33 h of durability tests, the voltage output of the triboelectric pairs stays relatively stable. Triboelectric outputs are affected by the frequency and pressure of contact–separation. Regarding the biological properties, these materials are cyto/hemocompatible, which supports the promising features of pHEMA and pHEMA/GO to develop biocompatible, hemocompatible, and biostable TENGs.

## Supporting Information Available

Graphene oxide characterization: elemental composition (atomic %) and C 1s high-resolution spectrum obtained by XPS; TEM image; and ζ potential (PDF)
