# Supplementary material for: Tailoring the Electron Trapping Effect of a Biocompatible Triboelectric Hydrogel by Graphene Oxide Incorporation towards Self-Powered Medical Electronics
Source: ACS Biomater Sci Eng. 2023 May 31;9(6):3712–22. doi: 10.1021/acsbiomaterials.2c01513 (PMC10265651; doi:10.1021/acsbiomaterials.2c01513)
Supplement: Supplementary file 1 — ab2c01513_si_001.pdf [file ab2c01513_si_001.pdf]

Supporting Information

Tailoring the electron trapping effect of a biocompatible triboelectric hydrogel by graphene oxide incorporation towards self-powered medical electronics

Andreia T. Pereira[1,2,#], Cátia R. S. Rodrigues[3,#], Ana C. Silva[3], Ricardo Vidal[1,2], João O. Ventura[3], Inês C. Gonçalves[1,2,], André M. Pereira[3],*

[1]i3S – Instituto de Investigação e Inovação em Saúde, Universidade do Porto, 4200-135 Porto, Portugal;
[2]INEB – Instituto de Engenharia Biomédica, Universidade do Porto, 4200-135 Porto, Portugal;
[3]IFIMUP – Instituto de Fisica de Materiais Avançados, Nanotecnologias e Fotónica, Departamento de Física e Astronomia, Faculdade de Ciências, Universidade do Porto, 4169-007 Porto, Portugal;

# the authors contribute equally
*ampereira@fc.up.pt

SI content

Number of Figures: 1 | Number of Pages: 2

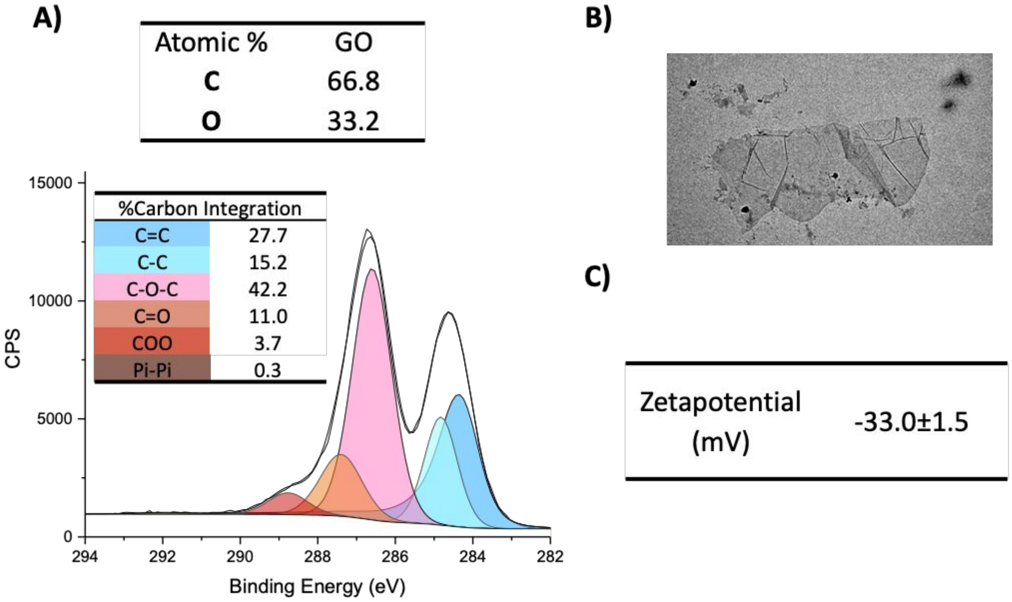

Figure S1. Graphene oxide characterization. A) elemental composition (atomic %) and C 1s high-resolution spectrum obtained by XPS, B) TEM image and C) Zeta potential
